# Effect of a Multicomponent Food Pantry Intervention in Client Subgroups

**DOI:** 10.3390/nu16060805

**Published:** 2024-03-12

**Authors:** Jenny Jia, Maria F. Gombi-Vaca, Christina Bliss Barsness, Hikaru Peterson, Rebekah Pratt, Julian Wolfson, Caitlin E. Caspi

**Affiliations:** 1 Division of General Internal Medicine, Department of Medicine, Northwestern University, Chicago, IL 60611, USA; 2 Rudd Center for Food Policy and Health, University of Connecticut, Hartford, CT 06103, USA; maria.gombi@uconn.edu (M.F.G.-V.); caitlin.caspi@uconn.edu (C.E.C.); 3 Department of Family Medicine and Community Health, University of Minnesota, Minneapolis, MN 55455, USArjpratt@umn.edu (R.P.); 4 Department of Applied Economics, University of Minnesota, St. Paul, MN 55108, USA; hhp@umn.edu; 5 Division of Biostatistics, University of Minnesota, Minneapolis, MN 55455, USA; julianw@umn.edu; 6 Department of Allied Health Sciences, University of Connecticut, Storrs, CT 06269, USA

**Keywords:** food pantries, food insecurity, diet quality, behavioral economics, nudge interventions, health equity

## Abstract

Nutrition promotion programs may have varying effects and influence health disparities. SuperShelf promotes healthy choices in food pantries through inventory changes and nudge implementation (e.g., choice architecture). This secondary analysis of the SuperShelf cluster-randomized trial assessed whether the effect of SuperShelf on client diet quality differed by equity characteristics. English-, Spanish-, or Somali-speaking adult clients from 11 food pantries in Minnesota were included (N = 193). We measured change in diet quality by the Healthy Eating Index 2015 (HEI-2015; maximum score 100) using up to two 24 h dietary recalls from pre-intervention and post-intervention periods. We used linear mixed-effects models to determine whether the effect of SuperShelf on diet quality varied by self-reported gender, race/ethnicity, education, and employment status. In separate adjusted models, the interactions of SuperShelf and gender, education, or employment status were not significant. The interaction of SuperShelf and race/ethnicity was significant (*p*-interaction = 0.008), but pairwise comparisons in diet quality were non-significant in all racial/ethnic subgroups. SuperShelf did not have differential effects on diet quality by gender, race/ethnicity, education, or employment status, suggesting it does not worsen dietary disparities among food pantry clients, though more subgroup analyses are needed to explore potential racial/ethnic disparities in this context.

## 1. Introduction

In the US in 2020, food pantries served free groceries to an estimated 60 million clients [1]. They are often reliable sources of healthier foods and strategic partners for disease prevention in vulnerable populations [2,3]. Recent pantry-based nutrition interventions have addressed client education, pantry environments, and pantry inventories [2,4,5].

Interventions in food pantries to “nudge” clients towards healthier food choices include choice architecture, where healthy foods are placed prominently, simplified labels to indicate healthfulness (e.g., stoplight green/yellow/red colors), and grocery bundles pre-packed for a healthy recipe. These nudges applied to food pantry environments have successfully led clients to choose healthier foods [5,6,7,8,9]. One study that implemented choice architecture and stoplight labels increased green food selection by 11% and reduced red food selection by 7% [9]. Another study of a diabetes-friendly food shelf with promotional posters found that clients chose more items from the intervention shelf (32–47%) compared to the general shelf (25–45%) over 6 months [7]. A study of healthy food bundles with a recipe at a food pantry demonstrated increased selection of healthy foods, compared to recipes alone and no intervention [8]. However, food insecurity and low socioeconomic status (SES) also affect decision making [10]. Though food pantry clients endorse preferring healthy foods [11,12,13], food insecurity and low SES can increase cognitive attention towards evading hunger, swaying clients away from preferred foods and long-term needs [14,15]. This maladaptive pressure may also vary by SES, highlighting the need for equity analyses in food pantry nutrition programs.

Understanding whether interventions promote or worsen health equity is important in nudge interventions without opt-outs, especially in vulnerable populations [16]. Prior studies show that some nudge interventions can inadvertently exacerbate health disparities [17,18]. In particular, certain types of nutrition labels favor higher-advantage groups, such as calorie labels [17,19]. Evidence on the equity of choice architecture interventions is more promising; one evaluation of pre-packed fruits and vegetables near checkout in three grocery stores led to more purchasing of the pre-packs by recipients of the Supplemental Nutritional Assistance Program (SNAP), compared to non-SNAP participants [20]. However, no prior equity evaluations exist for nudge interventions in food pantries.

SuperShelf was a site-randomized evaluation of a food pantry intervention to improve healthy food inventory and promote healthy choices [21]. To encourage clients to choose and consume healthier foods, the SuperShelf team worked with food pantries to maximize healthy sourcing to increase availability of healthy groceries and applied choice architecture to pantry shopping spaces to increase accessibility and visibility of healthy groceries. Unlike prior studies of nudge interventions in food pantries, the SuperShelf trial assessed change in both client food selection and client diet quality. Overall, SuperShelf did not significantly improve diet quality in pantry clients. Whether this null finding masks unequal effects in subgroups is unknown and important to mitigating disparities in pantry clients. In this analysis, we examined whether the effect of SuperShelf on diet quality was equitable by gender, race/ethnicity, education, and employment.

## 2. Materials and Methods

### 2.1. Study Population

This secondary analysis used data from the SuperShelf group-randomized, controlled trial (NCT03421106) [21]. Eligible food pantries were full “client-choice”, meaning clients could select any foods from the food pantry, akin to commercial stores, had staffing for SuperShelf activities, and were in Minnesota. The trial included 16 sites, matched in pairs by urbanization score [22]. Eligible adults were age 18 years or older, received groceries from the food pantry that day, had consistent access to a telephone, and spoke either English, Spanish, or Somali. Baseline data were collected February 2018 to June 2019, but only 11 sites completed follow-up data collection due to disruptions from the COVID-19 pandemic at 5 sites [23]. This study was approved by the [Redacted] Institutional Review Board. Further details of trial design and sampling were previously reported [23].

### 2.2. Intervention

SuperShelf transformed food pantries to promote healthy food choices in two phases [22]. First, SuperShelf-trained consultants worked with pantry staff and food bank representatives to implement strategies to increase procurement of healthy and culturally meaningful foods. Strategies included maximizing no-/low-cost healthy food sources (e.g., The Emergency Food Assistance Program, food rescue), developing new partnerships (e.g., local farms, community gardens), and messaging to increase healthier community donations.

Secondly, consultants and pantry staff reorganized food using behavioral economic strategies in client-accessible spaces to nudge clients towards healthier options. Strategies included food groups organized to have clients come across healthier food groups (e.g., fruits and vegetables) before less healthy ones (e.g., snacks, beverages, desserts), placing healthier options prominently (e.g., whole grains at eye level in the grains section, attractive displays), and bundling ingredients for a healthy recipe.

### 2.3. Measures

Dimensions of equity for this analysis were guided by the PROGRESS-Plus framework, which identifies factors associated with health inequities recommended by the Campbell and Cochrane Equity Methods Group [24,25]. Data were self-reported from the SuperShelf baseline survey: gender (female, male, non-binary), race/ethnicity (Hispanic/Latinx, Non-Hispanic [NH] Black, NH Native American/Alaskan Native, NH White, Additional Races), education (less than high school, high school diploma or equivalent, some college/associates/technical degree, four-year college degree and higher), and employment (currently employed, not employed). The Additional Races classification comprised groups with small sample sizes: multiracial, Asian, Native Hawaiian, write-in, and ‘prefer not to answer’ responses.

We measured client diet quality using the Healthy Eating Index 2015 (HEI-2015). The HEI-2015 had a maximum score of 100 with higher scores indicating healthier diet quality, based off how aligned one’s dietary intake is with the 2015–2020 Dietary Guidelines from Americans [26]. Clients completed up to two 24 h diet recalls by phone at baseline and 12-month follow up. HEI-2015 scores were generated using data from at least one 24 h dietary recall processed through a SAS macro created by the National Cancer Institute (“Simple HEI Scoring Algorithm—Per Day”).

### 2.4. Statistical Analysis

We generated descriptive statistics as frequencies with percentages for categorical variables and means with standard deviations for continuous variables. We used linear mixed-effects models to conduct difference-in-differences analyses to assess the association between participant intervention arm and change in participant HEI-2015 score from baseline to follow up. The models included fixed effects for treatment arm and timepoint (baseline or post-intervention), an interaction term between treatment and time, and random effects for sites and clients to account for correlated data. Models were adjusted for age group (18 to 44 years old, 45 to 64 years old, ≥65 years old), household composition (children in household, no children), frequency of food pantry visits (<1 visit per month, ≥1 visit per month), food from pantry in last 6 months (more than half of the food, less than half of the food), and baseline food pantry characteristics. Site characteristics included urban/rural status assigned by Rural–Urban Commuting Area code classifications and monthly pounds of food distributed, which was self-reported from food pantries. The models were fit via the maximum likelihood (ML) method, and the structure of the covariance matrix for the random effects was independent, which allows for a distinct variance for each random effect within a random-effects equation and assumes that all covariances are 0. We estimated intervention effects by each equity dimension using a 3-way interaction term between treatment, time, and equity variable and included all other equity variables as covariates in separate models. The test for heterogeneity determined whether the point estimates for any one subgroup was significantly different from another. We excluded 8 participants (4.2%) from the adjusted analysis due to missing covariate data. Analyses were performed in STATA, version 17, with statistical significance set at α = 0.05.

## 3. Results

This secondary analysis included 193 clients from 11 food pantries, of which 89 were in the intervention group, and 104 were in the control group (Table 1). At baseline, 122 (63.2%) identified as female, 69 (35.8%) as male, and 2 (1.0%) as non-binary. Race/ethnicity included 19 (9.8%) Hispanic/Latinx, 37 (19.2%) NH Black, 7 (3.6%) NH Native American/Alaskan Native, 118 (61.1%) NH White, and 12 (6.2%) Additional Races participants. Education included 22 (11.6%) with some high school or lower, 74 (39.2%) with a high school diploma or equivalent, 69 (36.5%) with some college or an associate or technical degree, and 24 (12.7%) with a college degree or higher. Finally, 62 adults (33.0%) were currently employed.

We found no significant intervention effect on HEI-2015 in all racial/ethnic subgroups (Table 2). The test for heterogeneity across racial/ethnic subgroups was statistically significant in both unadjusted and adjusted models (p-interaction = 0.008 and 0.005, respectively), but pairwise comparisons showed no significant differences (Table S1). In employed participants, the intervention group had a significantly higher HEI-2015 (difference 7.8, *p* = 0.041) than the control in the unadjusted model, but the difference was attenuated and nonsignificant in the adjusted model. We found no significant intervention effect on HEI-2015 in all gender, education, and employment subgroups, and the test for heterogeneity between these groups was not significant in both unadjusted and adjusted models (Table 2).

## 4. Discussion

In this secondary analysis of the SuperShelf group randomized trial, the effect of a multicomponent intervention focused on healthy food inventory and nudges on diet quality did not differ by gender, race/ethnicity, education, or employment, suggesting that SuperShelf was neutral towards health equity. No interactions were found between the intervention and gender, education, or employment on client diet quality, and no changes in diet quality were seen in any of the subgroups. While an interaction was statistically significant between SuperShelf and race/ethnicity, estimates of change in client diet quality within racial/ethnic subgroups were all non-significant. Because race and ethnicity are social constructs, the race/ethnicity measure is a reflection of other social exposures that could moderate intervention effects, such as socio-cultural differences [25] or varying risk of food insecurity [27]. Such factors may be driving small differences between racial/ethnic subgroups in SuperShelf, though study estimates were not precise enough to show definitive differences. Future food pantry interventions should consider culturally relevant choices and messaging. While this study generally did not find evidence that SuperShelf exacerbates disparities, future program evaluations in food pantries should continue to explore whether racial/ethnic disparities exist to better understand how its dimensions may influence program design and effectiveness among clients of food pantries.

Our finding that SuperShelf was neutral towards health disparities is consistent with prior studies of dietary nudges in different contexts. Studies of healthy food nudges in commercial settings suggest that such interventions are often neutral towards or can reduce health disparities [28,29]. A prior review of healthy eating interventions found that upstream interventions (e.g., price and environmental changes) generally reduced inequalities while downstream ones (e.g., individual education) increased inequalities [28]. Another review found that most studies of healthy food nudges demonstrated neutral effects or better healthy food selection in the more disadvantaged groups [29]. This study adds to the prior evidence by suggesting that healthy food choice nudges in a new setting, charitable food pantries, are neutral towards health disparities.

In commercial settings with general consumers, the ideal result of an equity study is that the intervention effect is greater in the lower equity group than the higher equity group as this promotes health equity [28]. Because the SuperShelf group-randomized study enrolled individuals who used food pantries, our sample was more representative of disadvantaged individuals than a general population. Hence, in equity analyses restricted to a lower equity sample, such as food pantry clients, interventions that have equal effects between lower and higher equity groups may be more ideal. This ensures that the intervention effect is not modified by degree of disadvantage in populations where disadvantage is prevalent.

While SuperShelf did not change diet quality in clients, interventions like SuperShelf may be more acceptable among clients since nudges promote healthy food selection without limiting choices [14]. Qualitative data from pantry staff in the SuperShelf trial suggested that SuperShelf had improved client experience, including increased availability of healthy choices; fewer negative client–staff encounters; and reduced stigma. These insights demonstrate the potential of interventions like SuperShelf to support a positive client experience. Future pantry programs should consider incorporating components of SuperShelf or pairing it with other interventions to promote health and improve client experience. Research on developing effective and acceptable nudge interventions for pantry clients is needed and may require greater community engagement with pantry clients and staff, partnership building with other health-promoting resources, and defining the role of food pantries within the greater mission to improve health equity. Finally, the decision to implement programs like SuperShelf should also consider community need and organizational capacity, highlighting the need for research on the implementation context of food pantries and what organization characteristics correlate with successful implementation.

This secondary analysis has limitations. The main SuperShelf sample was not sufficiently powered to conduct this equity analysis, which limited our ability to detect effects within subgroups and differences in effects between subgroups. This is a common limitation in equity analyses, and future studies should consider complex sampling techniques for pre-planned equity analyses. Stratification in this secondary analysis of randomized data also likely led to confounding since the initial randomization scheme did not consider equity dimensions, which was mitigated using adjusted models. Furthermore, we combined small sample sizes with certain racial groups (multiracial, Asian, Native Hawaiian, write-in, and prefer not to answer) into “Additional Races”, which does not represent an underlying identity and likely has high heterogeneity. Combining these subgroups might obscure patterns that may exist and does not allow us to identify their specific needs or concerns. Finally, participants likely had varying levels of exposure to SuperShelf as some rarely visited the food pantry or stopped going altogether during the study, which may have biased results towards the null.

## 5. Conclusions

SuperShelf, an intervention to transform food pantry environments through healthy food inventory and nudges, is likely neutral towards health equity in underserved adults, though additional studies on the influence of race/ethnicity in food pantry nutrition programs are necessary. SuperShelf did not clearly affect diet quality in any subgroup of gender, race/ethnicity, education, or employment status. Behavioral economic interventions have potential to balance health equity and individual choice, but further research on increasing their effectiveness in food pantries is needed, including community-engaged intervention codesign, health promotion partnerships, and implementation strategies.

## Figures and Tables

**Table 1:** Client demographic, socioeconomic, and food pantry characteristics with HEI-2015 scores at baseline.

	Intervention			Control		
	n = 87	Baseline HEI-2015 (n = 87)		n = 104	Baseline HEI-2015 (n = 103)	
	n (%)	Mean (SD)	*p*-Values ^1^	n (%)	Mean (SD)	*p*-Values ^1^
Age group (years)						
18–44	37 (41.6)	49.8 (15.8)	0.96	39 (37.5)	48.5 (14.7)	0.01
45–64	37 (41.6)	50.8 (15.2)		52 (50.0)	47.4 (14.7)	
65+	15 (16.9)	50.2 (14.4)		13 (12.5)	61.3 (13.5)	
Gender						
Female	57 (64.0)	49.7 (14.4)	0.62	65 (64.4)	50.9 (15.5)	0.07
Male	32 (36.0)	51.4 (16.6)		36 (35.6)	45.4 (12.0)	
Non-binary/prefer not to answer				2 (1.9)	81.6 (5.4)	
Race/ethnicity						
Hispanic/Latinx	10 (11.2)	63.5 (10.6)	0.04	9 (8.7)	61.5 (14.9)	0.04
NH Black	9 (10.1)	45.9 (11.8)		28 (26.9)	46.2 (10.2)	
NH Native American/Alaskan Native	4 (4.5)	54.2 (18.9)		3 (2.9)	40.1 (12.3)	
NH White	60 (67.4)	48.9 (14.7)		58 (55.8)	49.0 (16.7)	
Additional Races ^2^	6 (6.7)	45.8 (20.5)		6 (5.8)	57.2 (7.9)	
Primary language						
English	82 (92.1)	48.7 (14.6)	0.001	102 (98.1)	49.3 (15.0)	0.19
Other	7 (7.9)	68.2 (8.6)		2 (1.9)	63.4 (20.5)	
Education						
≤Some high school	8 (9.1)	46.5 (12.8)	0.06	14 (13.9)	51.9 (12.8)	0.56
HS diploma or GED	31 (35.2)	49.1 (14.8)		43 (42.6)	48.0 (14.5)	
Technical/associates degree or some college	32 (36.4)	47.5 (13.8)		37 (36.6)	47.6 (14.6)	
≥4-year college degree	17 (19.3)	59.2 (17.3)		7 (6.9)	54.7 (18.7)	
Children in household						
Yes	36 (40.5)	49.2 (12.4)	0.60	44 (42.3)	51.0 (16.3)	0.41
No	53 (59.6)	51.0 (16.9)		60 (57.7)	48.5 (14.2)	
Employment						
Currently employed	33 (37.9)	46.9 (14.0)	0.13	29 (28.7)	47.5 (15.7)	0.54
Not employed	54 (62.1)	52.1 (15.8)		72 (71.3)	49.5 (14.2)	
Food pantry						
<1 visit per month	22 (22.6)	47.1 (16.4)	0.22	26 (25.2)	44.7 (10.2)	0.06
≥1 visit per month	64 (74.4)	51.6 (14.5)		77 (74.8)	50.8 (15.7)	
Food from pantry in the last 6 months						
More than half of the food	42 (47.2)	51.3 (12.7)	0.56	46 (44.7)	49.1 (13.1)	0.93
Less than half of the pantry	47 (52.8)	49.4 (17.1)		57 (55.3)	49.4 (16.0)	
Food pantry location						
Urban	57 (64.0)	50.4 (13.9)	0.94	54 (51.9)	49.7 (13.3)	0.92
Rural	32 (36.0)	50.1 (17.3)		50 (48.1)	49.4 (16.9)	

^1^ *p*-values measure heterogeneity in HEI-2015 scores by between levels of each covariate; ^2^ Comprised groups with small sample sizes: multiracial, Asian, Native Hawaiian, write-in, and prefer not to answer.

**Table 2:** Difference-in-differences in Healthy Eating Index 2015 Scores by Health Equity Measures.

	Baseline HEI-2015 Mean (SD)	Unadj. Difference-in-Difference (95% CI)	*p*-Value	Test for Heterogeneity	Adj. Difference-in-Difference (95% CI)	*p*-Value	Test for Heterogeneity
Gender							
Female	50.3 (15.0)	2.6 (−2.4, 7.5)	0.309	0.331	2.2 (−3.1, 7.5)	0.412	0.410
Male	48.2 (14.5)	0.3 (−6.7, 7.3)	0.934		−0.6 (−7.8, 6.6)	0.872	
Race/ethnicity							
Hispanic/Latinx	62.5 (12.5)	2.6 (−10.0, 15.1)	0.688	0.008	−1.7 (−15.5, 12.1)	0.813	0.005
NH Black	46.2 (10.5)	−3.0 (−13.4, 7.3)	0.564		−4.2 (−15.0, 6.6)	0.444	
NH Native American/Alaskan Native	48.1 (16.9)	−6.0 (−30.0, 18.1)	0.627		−6.3 (−29.9, 17.3)	0.600	
NH White	48.9 (15.7)	3.8 (−1.5, 9.1)	0.160		3.0 (−2.4, 8.3)	0.275	
Additional Races ^1^	51.5 (16.0)	7.2 (−10.0, 24.4)	0.412		5.8 (−12.3, 23.9)	0.529	
Education level							
Some HS or less	50.0 (12.7)	1.1 (−11.2, 13.5)	0.856	0.119	2.7 (−10.1, 15.6)	0.676	0.097
HS diploma or equivalent	48.5 (14.5)	6.6 (−0.4, 13.5)	0.064		6.5 (−0.8, 13.8)	0.080	
Technical/associates degree or some college	47.6 (14.1)	2.1 (−4.4, 8.6)	0.524		0.9 (−5.7, 7.5)	0.790	
4-year college degree or higher	57.9 (17.5)	−7.2 (−19.9, 5.4)	0.263		−8.8 (−21.6, 3.9)	0.173	
Employment							
Currently employed	47.2 (14.7)	7.8 (0.3, 15.3)	0.041	0.213	7.3 (−0.5, 15.1)	0.067	0.251
Not employed	50.6 (14.9)	−0.2 (−5.2, 4.7)	0.931		−1.4 (−6.5, 3.7)	0.594	

^1^ Comprised groups with small sample sizes: multiracial, Asian, Native Hawaiian, write-in, and prefer not to answer.

## Data Availability

Raw data will be retained and are readily available if requested.

## Supplementary Materials

The following supporting information can be downloaded at: https://www.mdpi.com/article/10.3390/nu16060805/s1, Table S1: Pairwise comparisons of adjusted difference-in-differences in HEI-2015 by subgroups of race/ethnicity.
