# Supplementary material for: Effect of a Multicomponent Food Pantry Intervention in Client Subgroups
Source: Nutrients. 2024 Mar 12;16(6):805. doi: 10.3390/nu16060805 (PMC10974327; doi:10.3390/nu16060805)
Supplement: Supplementary file 1 [file nutrients-16-00805-s001.zip › nutrients-2857666-supplementary.pdf]

Table S1. Pairwise comparisons of adjusted difference-in-differences in HEI-2015 by subgroups of race/ethnicity

	Hispanic/ Latinx	NH Black	NH Native American/ Alaskan Native	NH White	Additional race or missing[1]
			versus		
Hispanic/Latinx	-	-2.5	-4.6	4.6	7.5
NH Black	2.5	-	-2.1	7.2	10.0
NH Native American/Alaskan Native	4.6	2.1	-	9.3	12.1
NH White	-4.6	-7.2	-9.3	-	2.9
Additional Races[1]	-7.5	-10.0	-12.1	-2.9	-

All pairwise comparisons have p-value>0.05.

[1] Comprised of groups with small sample sizes: multiracial, Asian, Native Hawaiian, write-in, and prefer not to answer
